# PREDICTORS AND IMPLICATIONS OF GENERATIVITY AMONG LGBTQ+ OLDER ADULTS

**DOI:** 10.1093/geroni/igae098.2194

**Published:** 2024-12-31

**Authors:** Hailey Jung, Hyun-Jun Kim, Charles Emlet, Karen Fredriksen-Goldsen

**Affiliations:** University of Washington, Seattle, Washington, United States; University of Washington, Seattle, Washington, United States; University of Washington Tacoma, Tacoma, Washington, United States; University of Washington, Seattle, Washington, United States

## Abstract

Generativity is a concern, commitment, and action for others including the next generation, and has been shown positively associated with the health and well-being of older adults. Since LGBTQ+ older adults have experienced overt marginalization and often have limited contact with younger generations, generativity may resonate with unique implications in LGBTQ+ communities. Yet, to date research on generativity with this community is lacking. In this paper, we integrate Generativity Theory and Health Equity Promotional Model (HEPM) to examine the predictors of generativity among LGBTQ+ older adults and explore key psychological and social factors that moderate the relationship between stigma experiences and generativity. Using the 2016 data from Aging with Pride: National Health, Aging, and Sexuality/Gender Study (N = 2,233), we regressed generativity in three blocks; first, social location and stigma variables, next, psychological resources, and lastly, social resources. The predictive power of the model significantly improved in each step. Psychological resources (i.e., mastery, resilience, and critical awareness) and social resources, (i.e., social participation, network size, social support, and LGBTQ+ community engagement) were all positively associated with generativity. In terms of moderating effects, day-to-day discrimination no longer had negative effects on generativity when experienced by older adults with higher levels of critical awareness. Interestingly, lifetime discrimination had positive effects on generativity for individuals with higher mastery levels. Findings suggest psychological and social mechanisms that may foster fortitude and stress-related growth among LGBTQ+ older adults. This research provides valuable insights to foster intergenerational relationships and cultivate strengths in the LGBTQ+ community.

